# Stingless Bee (*Heterotrigona Itama*) Honey and Its Phenolic-Rich Extract Ameliorate Oxidant–Antioxidant Balance via KEAP1-NRF2 Signalling Pathway

**DOI:** 10.3390/nu15132835

**Published:** 2023-06-22

**Authors:** Mohamad Zulhafiz Shafiq Zulhilmi Cheng, Fatin Aina Zulkhairi Amin, Norhasnida Zawawi, Kim Wei Chan, Norsharina Ismail, Nur Akmal Ishak, Norhaizan Mohd Esa

**Affiliations:** 1 Natural Medicines and Products Research Laboratory, Institute of Bioscience, Universiti Putra Malaysia, Serdang 43400, Selangor, Malaysia; 2 Department of Food Science, Faculty of Food Science and Technology, Universiti Putra Malaysia, Serdang 43400, Selangor, Malaysia; 3 Center of Foundation Studies for Agricultural Science, Universiti Putra Malaysia, Serdang 43400, Selangor, Malaysia; 4 Department of Nutrition, Faculty of Medicine and Health Sciences, Universiti Putra Malaysia, Serdang 43400, Selangor, Malaysia

**Keywords:** stingless bee honey, phenolic-rich extract, oxidative stress, KEAP1-NRF2 signalling

## Abstract

Diabetes is associated with an imbalance between oxidants and antioxidants, leading to oxidative stress. This imbalance contributes to the development and progression of diabetic complications. Similarly, renal and liver diseases are characterised by oxidative stress, where an excess of oxidants overwhelms the antioxidant defense mechanisms, causing tissue damage and dysfunction. Restoring the oxidant–antioxidant balance is essential for mitigating oxidative stress-related damage under these conditions. In this current study, the efficacy of stingless bee honey (SBH) and its phenolic-rich extract (PRE) in controlling the oxidant–antioxidant balance in high-fat diet- and streptozotocin/nicotinamide-induced diabetic rats was investigated. The administration of SBH and PRE improved systemic antioxidant defense and oxidative stress-related measures without compromising liver and renal functioning. Analyses of the liver, skeletal muscle and adipose tissues revealed differences in their capacities to scavenge free radicals and halt lipid peroxidation. Transcriptional alterations hypothesised tissue-specific control of KEAP1-NRF2 signalling by upregulation of *Nrf2, Ho1* and *Sod1* in a tissue-specific manner. In addition, hepatic translational studies demonstrated the stimulation of downstream antioxidant-related protein with upregulated expression of SOD-1 and HOD-1 protein. Overall, the results indicated that PRE and SBH can be exploited to restore the oxidant–antioxidant imbalance generated by diabetes via regulating the KEAP1-NRF2 signalling pathway.

## 1. Introduction

The pathogenic factor that increased the risk of type 2 diabetes mellitus (T2DM) has been linked to changes in oxidative stress and antioxidant defence. Hyperglycemia causes free radical formation by inducing non-enzymatic glycosylation and glucose auto-oxidation. Abnormal levels of free radicals overwhelm the body’s natural antioxidant defences, leading to insulin resistance [1]. In addition, the oxidant–antioxidant imbalance results in hepatic and renal diseases, which are closely associated with oxidative stress [2,3]. Phenolic is a compound derived from natural sources and is associated with beneficial health effects due to its antioxidant properties [4].

Free radicals are highly reactive molecules that can damage cells and contribute to the development of chronic diseases such as cancer, diabetes, and cardiovascular disease [5]. Antioxidants protect the body against free radicals by converting them into less harmful molecules, which stop the radical chain reaction that causes oxidation [6,7]. This process helps prevent the accumulation of oxidative damage in cells and tissues, which can lead to the development of various diseases. Several studies have shown that natural antioxidants can prevent different types of diseases, including diabetes mellitus, cancer and hypertension [8,9,10]. An antioxidant has two direct action modes: a preventive antioxidant and a chain-breaking antioxidant, which scavenges free radicals to alleviate disease [11]. The importance of the kelch-like ECH-associated protein 1 (KEAP1)–nuclear factor, erythroid 2-like 2 (NRF2) pathways in various physiological and pathological processes, especially in energy metabolism, has piqued researchers’ curiosity. The transcription of genes that bind active NRF2 activates the code for antioxidant enzymes to the relevant antioxidant-responsive regions in those genes [12]. Since it plays a part in maintaining redox balance in the body and insulin action, it can also serve as a therapeutic target in treating diabetes [13].

Stingless bee honey (SBH) from *Heterotrigona itama* contains a high concentration of polyphenols (phenolic acids, flavonoids and their derivatives), which are thought to contribute to honey’s strong antioxidant activity [14,15]. The phenolic-rich extract (PRE) of SBH obtained by solid-phase extraction is rich in polyphenolic content and has shown potential as an antioxidant therapeutic agent in vitro [16]. The current study sought to present novel insights into the potential therapeutic application of SBH and its PRE for managing oxidative stress and restoring the oxidant–antioxidant balance in diabetes by investigating the systemic antioxidative effects in high-fat diet streptozotocin/nicotinamide-induced diabetic rats. The tissue-specific antioxidant properties with translational patterns of essential genes associated with the KEAP1-NRF2 signalling pathways were analysed. PRE and SBH were also examined for their impact on liver and renal function.

## 2. Materials and Methods

### 2.1. Chemical and Reagent

Chemi-Lumi One L, phosphate-buffered saline (PBS), RIPA buffer, and protease and phosphatase inhibitor cocktails were acquired from Nacalai Tesque (Kyoto, Japan). The standard rat pellet was obtained from Gold Coin, Singapore. Roche Diagnostics was obtained for Accu-Check blood glucose test strips (Indianapolis, IN, USA). Elisa kits, 8-oxo-2′-deoxyguanosine and 8-isoprostaglandin F2α were purchased from ElabScience (Houston, TX, USA). RNAlaterTM, TRIzol and proteinase-K were purchased from ThermoFisher (Waltham, MA, USA). The total RNA Isolation kit was manufactured by RBC Biocorp (Taipei, Taiwan). Kits for measuring lipid profiles, liver and kidney function, as well as glutathione peroxidase (GPx) and superoxide dismutase (SOD), were acquired from Randox Laboratories Ltd. (Crumlin, Country Antrim, UK). The following chemicals were purchased from Sigma Chemical Co.: Ethylenediaminetetraacetic acid (EDTA), 2,2′-azino-bis (3-ethylbenzthiazoline-6-sulphonic acid) (ABTS), 2,2′-Azobis (2-amidinopropyl) dihydrochloride (AAPH), hydrogen peroxide (H_2_O_2_), trichloroacetic acid (TCA) and tetra-methylchroman-2-carboxylic acid (Trolox) (St. Louis, MI, USA). Protein A/G PLUS-Agarose was purchased from Santa Cruz Biotechnology, Inc. (Dallas, TX, USA). Millipore supplied the 0.45 m Immobilon-FL polyvinylidene fluoride (PVDF) membrane (Bedford, MA, USA). ABClonal Technology supplied superoxide dismutase 1 (SOD1), heme oxygenase 1 (HO1), nuclear factor erythroid 2–related factor 2 (NRF2), glyceraldehyde-3-phosphate dehydrogenase (GAPDH) and HRP-conjugated goat anti-rabbit secondary antibodies (Woburn, MA, USA).

### 2.2. Preparation of Stingless Bee Honey (SBH) and Phenolic-Rich Extract (PRE)

Stingless bee (*Heterotrigona itama*) honey (SBH) samples were freshly harvested from sealed stingless bee honey pots in Sibu, Malaysia (2°41′38.3″ N 111°41′03.3″ E) in July 2017. The collected honey samples were kept in sterilised bottles at 4 °C for further analysis.

An SPE Agilent Bond Elut C18 column (3 mL × 1 g) (Agilent, Santa Clara, CA, USA) was used for the preparation of PRE from SBH [17] with a slight adjustment. Briefly, 2 g of SBH was diluted with 100 mL of acidified water (0.5% orthophosphoric acid). The SPE C18 column was preconditioned with 5 mL of methanol, followed by 5 mL of acidified water. The column was then loaded with 5 mL of diluted SBH sample, eluted with 5 mL of 90% methanol, and the PRE yielded was dried at 40 °C for 24 h.

### 2.3. Experimental Animal

Sprague Dawley (SD) rats were provided and supplied by A-Saphhire Enterprise, Jalan Indah 2/8, Taman Universiti Indah, 43300 Seri Kembangan, Selangor. The animals were handled in compliance with the institutional rules issued by the animal ethics committee. The Animal Ethic Committee of Universiti Putra Malaysia has accepted the protocol below (Ref: UPM/IACUC/2019/R008).

### 2.4. Induction of Diabetes

Male Sprague Dawley (SD) rats (*n* = 56, 6 weeks old, body weight 76.86 ± 7.73 g) were acclimatised for one week under controlled conditions (25 °C, 12/12 h light/dark cycle). Body weight was measured following the acclimatisation phase and then at weekly intervals. Post acclimatisation, the rats were subjected to a high-fat diet for four weeks to induce obesity.

The high-fat diet consisted of 40% carbohydrate, 30% protein, 22.5% fat and 2.5% for cholesterol, fiber and vitamin, respectively [18], whereas normal control (NC) rats were fed a standard pellet meal throughout the experiment. After four weeks on a high-fat diet, the average body weight of the high-fat diet group was 30.05% higher than that of the normal control (NC), which is defined as obese [19] (Supplementary Figure S1). Streptozotocin (STZ-60 mg/kg body weight) and nicotinamide (NAM-120 mg/kg body weight) were injected intraperitoneally into overnight-fasted obese rats at week five [18]. As a reference control, the rats fed with a regular diet were given normal saline and sodium citrate buffer (pH 4.5) instead of streptozotocin and nicotinamide. After one week, moderate hyperglycemia of 8.5 ± 0.91 mmol/L was reported in STZ-NAM-induced diabetic rats.

The diabetic rats were randomly divided into six groups (*n* = 8), and the intervention treatment outlined in Table 1 was carried out for 50 days via oral gavage. The rats consumed high-fat food during the treatment, except in the normal control group. At the end of the treatment, the rats that had fasted overnight were put to death by cardiac puncture using ketamine (100 mg/kg) + xylazine (10 mg/kg). A cardiac puncture technique was used to collect serum and whole blood samples. The liver, adipose and skeletal muscle tissues were collected and preserved in an RNAlaterTM stabilisation solution for future analysis.

### 2.5. Biochemical Analysis

#### Kidney and Liver Function

The levels of aspartate aminotransferase (AST), alanine aminotransferase (ALT), alkaline phosphatase (ALP), gamma glutamic transpeptidase (GGT), creatinine and urea were measured in the collected serum using a Selectra XL instrument (Vita Scientific, Dieren, The Netherlands) with Randox analytical kits in accordance with the instructions provided by the manufacturer.

### 2.6. Antioxidant Analysis

#### 2.6.1. Total Antioxidant Status (TAS)

Serum and tissue homogenates were evaluated for their total antioxidant status (TAS) based on their capacity to scavenge ABTS radical cations. The TAS assay was performed according to the method described by Chan et al. [20] with a slight adjustment. Tissue samples were homogenised in PBS and centrifuged at 10,000× *g* for 10 min at 4 °C to produce a 10% tissue homogenate (*w/v*). Quantification of protein contents was determined using bicinchoninic acid (BCA) protein assay kit. In a 96-well plate, 20 µL of suitably diluted material was mixed with 200 µL of the calibrated ABTS radical cation mixture to test its scavenging activity. After 10 min of incubation, spectrophotometric absorbance was measured at 734 nm. TAS was expressed as nmol Trolox equivalent (TRE)/mg protein, with Trolox as the positive control.

#### 2.6.2. Oxygen Radical Absorbance Capacity (ORAC)

Oxygen radical absorbance capacity (ORAC) was determined by evaluating the ability of samples to reduce free radical damage to the fluorescent probe [21]. This experiment used AAPH as the free-radical source and fluorescein as the fluorescent probe. Briefly, 25 µL of serum or tissue homogenates were mixed with 150 µL of 0.08 M fluorescein at 37 °C for 15 min. To commence the reaction, 25 µL of newly produced 153 mM AAPH was added. The kinetic fluorescence spectrum with excitation at 485 nm/em 528 nm was tracked for 2 h, with minute-by-minute readings recorded on a BioTek Synergy H1 Hybrid reader (BioTek Instruments Inc., Winooski, VT, USA). The area under the curve *(AUC)* and net *AUC* were computed as follows:

*AUC* = 0.5 + (*R*1/*R*0) + (*R*2/*R*0) + (*R*3/*R*0) + …… + 0.5 (*R*n/*R*0) (1)

*Net AUC = AUCs* − *AUC*0 (2)

where *R0* is the initial fluorescence measurement, *Rn* is the final reading, *AUC0* is the area under the curve for the blank, and *AUCs* is the area under the curve for the sample mixture. Trolox was utilised as the positive standard, and the ORAC values were represented as µmol Trolox equivalent (TRE)/mg protein.

#### 2.6.3. Thiobarbituric Acid Reactive Substances Assay (TBARS)

Serum and tissue homogenates were analysed for thiobarbituric acid reactive substance (TBARS) concentrations using a modified method described by Ooi et al. [22]. First, 200 µL of 10% (*w/v*) ice-cold trichloroacetic acid (TCA) was added to 100 µL of the sample. After 15 min on ice, the protein content was precipitated by centrifugation at 2200× *g* for 15 min at 4 °C. Then, 200 mL of the supernatant was collected, and an equal volume of 10% TCA (*w/v*) at 0.67 percent was added subsequently. The mixture was incubated for 10 min at 100 °C in a water bath to produce a pink chromogen. The BioTek Synergy H1 Hybrid reader captured the absorbance with excitation at 560 nm/em 585 nm (BioTek Instruments Inc., Winooski, VT, USA). Malondialdehyde (MDA) standard curves were generated using 1,1,3,3-tetramethoxy propane, and TBARS results were represented as nmol MDA/mg protein.

#### 2.6.4. Low Molecular Weight Advanced Glycation End Products (LMW-AGEs)

LMW-AGEs were measured using fluorometry, as previously described by Balamash et al. [23]. Briefly, 20 µL of blood samples were treated with 480 µL of ice-cold 0.15 M trichloroacetic acid and 100 µL of chloroform to precipitate the vast molecular weight protein and eliminate the lipid content. The fluorescence intensity of the aqueous phase with excitation at 370 nm/em 440 nm was measured using a BioTek Synergy H1 Hybrid reader after vortex mixing and centrifugation (BioTek Instruments Inc., Winooski, VT, USA). The results were represented in arbitrary units (AU)/mg protein.

#### 2.6.5. Antioxidant Enzymes and Oxidative Stress Markers

The antioxidant enzymes glutathione peroxidase (GPx) and superoxide dismutase (SOD) were measured in heparinised whole blood samples using Randox analytical kits according to the manufacturer’s instructions. Using respective Elisa kits, measurements of oxidative stress markers, such as 8-oxo-2′-deoxyguanosine (8-Ohdg) E-EL-0028 and 8-isoprostaglandin F2 (8-iso-Pgf2) E-EL-0041, were conducted in the serum that was collected.

### 2.7. Gene Expression Analysis

#### 2.7.1. RNA Extraction

A total RNA isolation kit-HiYield-YRT300 (RBC Biotech) was used to extract total RNA from the liver, adipose and muscle tissues, with minor modifications to the manufacturer’s instructions. Notably, TRIzol substituted the lysis buffer for the RNA extraction of adipose and muscle tissue. Briefly, 25 mg of tissue was dissolved in 400 µL of RB buffer and 4 µL of β-mercaptoethanol using a micro pestle and a 23 G needle syringe. The tissue was then incubated for 5 min before being transferred via a lysate filter column to a collection tube. The lysis phase was concluded with a 1 min centrifuge at 1000× *g*. For RNA binding, 400 µL of 70% ethanol was added to the filtrate before transferring it to another collecting tube via an RT column and centrifuged for 2 min at maximum speed. The filtrate was washed multiple times with W1 buffer (400 µL) and wash buffer (600 µL), and then dried by centrifuging at high speed for 5 min. Then, 50 µL of RNase-free water was used as the solvent and allowed to stand at room temperature for 5 min for optimal absorption before centrifuging the purified RNA at full speed for 1 min.

#### 2.7.2. cDNA Synthesis

Utilising the SensiFAST cDNA Synthesis Kit (BIO-65054), cDNA was synthesised for quantitative real-time PCR (qPCR) investigations. The sample tissue total RNA, 5× TransAmp buffer, RNA transcriptase, and RNase-free water were mixed to produce a 20 µL master mix. The master mix was then subjected to a thermal cycler with the following parameters: 25 °C for 10 min for primer annealing, 42 °C for 30 min for reverse transcription, 85 °C for 5 min for inactivation, and 4 °C hold.

#### 2.7.3. Quantitative Transcription Polymerase Chain Reaction (qPCR)

The SensiFAST^TM^ SYBR No-ROX Kit (BIO-98020) was used for qPCR analysis. The PCR master mix includes 2× SensiFAST SYBR No-ROX Mix, 10 µM forward primer, 10 µM reverse primer (Supplementary Table S1), DNase-free water, and a sample template. The 3-step cycling conditions were as follows: 95 °C for 2 min to activate the polymerase, 95 °C for 5 s to denature, 65 °C for 10 s to anneal, and 72 °C for 20 s to extend. Glyceraldehyde 3-phosphate dehydrogenase (GAPDH) was used as an internal control, and relative mRNA expression was determined in triplicates using the Livak method (2^−ΔΔCT^).

### 2.8. Western Blotting

The liver was homogenised in ice-cold RIPA lysis solution (Nacalai Tesque, Inc.) to produce total protein lysates, which contained protease and phosphatase inhibitor cocktails. The lysate was then centrifuged at 12,000× *g* for 20 min at 4 °C to obtain clear supernatants. The protein concentrations were determined using a PierceTM BCA protein assay kit. An equal concentration of proteins was separated on a 10% SDS-PAGE gel, transferred to a PVDF membrane, and probed with primary antibodies. The primary antibodies used were GAPDH-rabbit (1:5000; 36 kDa), NRF2 (1:1000; 97 kDa), SOD1 (1:1000; 16 kDa) and HO1 (1:1000; 32 kDa). For the detection of immunoreactive bands, HRP-conjugated goat anti-mouse and goat anti-rabbit secondary antibodies and Western Bright ECL were utilised. ChemiDoc MP System (Bio-Rad, Hercules, CA, USA) and ImageJ (Java) software were used for quantitative analyses. To assess the relative expression levels, the density of each target band was computed relative to the Gapdh protein band.

### 2.9. Statistical Analysis

Using GraphPad Prism 9 (San Diego, CA, USA), the statistical significance of differences was assessed using one-way ANOVA with Tukey’s test analysis, where *p* < 0.05 signifies a statistically significant difference. Spearman’s correlation was used to identify pairwise correlations between the antioxidant defence and relative mRNA expression of target genes.

## 3. Results and Discussion

### 3.1. Biochemical Analysis

#### Liver and Kidney Profile Function

The impact of SBH and PRE treatments on AST, ALT and ALP activities in the serum of normal and diabetes-induced rats is shown in Table 2. Under diabetic conditions, the antidiabetic treatment puts a substantial additional burden on the liver and kidneys for detoxification, which consequently affects both hepatic and renal metabolic processes. The results demonstrated that the AST and ALT activities in the serum of the DC group were significantly higher (*p* > 0.05) compared to the NC group. These enzymes are often abundant in the liver and are crucial for the metabolism of amino acids [24]. Nevertheless, these enzymes may drain from the hepatocytes into the bloodstream due to liver injury or toxicity, where their concentrations become high, indicating hepatocellular injury.

In addition, the findings revealed an increase in ALP activity in the serum of the DC group. ALP, primarily located in the liver’s bile ducts, is a sign of healthy bile flow, cholestasis and liver health [24,25]. There is a possibility that the increased levels of these enzymes were caused by the decreased levels of insulin found in STZ-induced diabetic rats [26]. In STZ-NAM-induced diabetic rats, SBH-HD, PRE-LD and PRE-HD significantly (*p* < 0.05) lowered the increased activities of AST, ALT and ALP. The result suggests that the treatment with SBH-HD and PRE has a hepatoprotective effect on diabetic rats induced by STZ-NAM. On the other hand, the SBH-LD group, which had a low antioxidant potential, did not stop the liver function from worsening in diabetic rats, as its AST and ALP readings did not differ much from those of the DC group (*p* > 0.05). These findings provide more evidence that supports the findings of other authors, who also showed elevated AST, ALT and ALP in STZ-induced diabetic rats [25,27].

The serum urea levels did not significantly change (*p* > 0.05) in any diabetes-induced groups. In the DC group, the blood levels of creatinine were elevated, indicating decreased renal function. Although significantly different, the levels of creatinine in both MC- and SBH-treated groups remained high. However, PRE-HD treatment dramatically reduced serum creatinine levels, favouring the avoidance of kidney damage. Furthermore, higher phenolic content and antioxidant capacity in PRE-HD were significant factors that may have been responsible for better outcomes for both liver and kidney function parameters.

### 3.2. Antioxidant Analysis

#### 3.2.1. Overall Antioxidant Defense

Re-establishing a healthy equilibrium between the body’s oxidant and antioxidant defences has been proposed as a potential treatment strategy for diabetes and diabetic complications. Reactive oxygen species (ROS) and oxidative stress are linked to elevated blood glucose levels and cause a persistent level of elevated blood glucose, which is hazardous to the cardiovascular system, a condition known as glucotoxicity [28,29].

**Figure 1:**
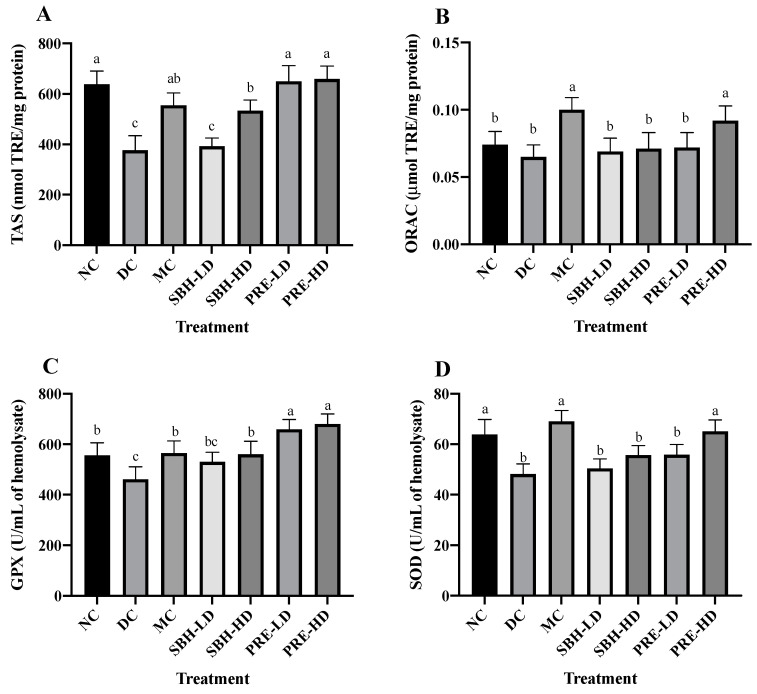
Overall antioxidant defense of the treatment groups. (**A**) TAS, (**B**) ORAC, (**C**) GPX and (**D**) SOD of the serum. The values are expressed as the mean ± standard deviation (*n* = 8); different letters indicate significant differences (*p* < 0.05). NC, normal group; DC, diabetic control group without treatment; MC, metformin-treated diabetic control; SBH-LD, diabetic rats treated with stingless bee honey (0.25 g/kg body weight); SBH-HD, diabetic rats treated with stingless bee honey (0.5 g/kg body weight); PRE-LD, diabetic rats treated with the phenolic-rich extract (12.5 mg/kg body weight); PRE-HD, diabetic rats treated with the phenolic-rich extract (25.0 mg/kg body weight).

The study investigated free radicals, ABTS radical cations and AAPH-derived peroxyl radicals, which were analysed using total antioxidant status (TAS) and oxygen radical absorbance capacity (ORAC) assays. For the TAS assay, diabetes-induced rats treated with PRE-HD showed the highest scavenging ability to eliminate ABTS radical cations by 75.4%, followed by PRE-LD (72.9%) and SBH-HD (42.0%) (*p* < 0.05) compared to the DC group (Figure 1). In contrast, only MC and PRE-HD were demonstrated to possess significant scavenging activities towards AAPH-derived peroxyl radical 1.5- and 1.4-fold (*p* < 0.05), respectively, compared to the DC group for ORAC.

The concentrations of antioxidant enzymes glutathione peroxidase (GPx) and superoxide dismutase (SOD) were quantified to investigate the nature of the system’s antioxidant defence. Compared to the DC group, the findings showed that the concentrations of GPx were significantly higher in both the PRE-HD and PRE-LD groups by 47.6% and 43.1%, respectively, which is statistically significant (*p* < 0.05). Although the GPx values in the MC group and SBH-HD group were significantly higher (*p* < 0.05) than those in the DC group, they were still significantly lower (*p* < 0.05) than those in the PRE treatment groups. The levels of superoxide dismutase (SOD) in the MC and PRE-HD groups were significantly greater than those in the DC group by 43.4% and 35.13%, respectively. According to Diniz Vilela et al. [30], metformin therapy decreases the protein levels of SOD, which indirectly diminishes the expression of these enzymes by reducing ROS generation and oxidative stress. Another in vivo study by Erejuwa et al. [31], Omatayo et al. [32] and Erejuwa et al. [33] regarding honey and antioxidant show that Tualang honey significantly increased TAS, GPx and SOD levels in diabetes-induced rats.

The results clearly indicated that PRE is a good alternative for antioxidant defence against free radicals and antioxidant enzymes. These results align with the in vitro antioxidant investigation [16] (Supplementary Figure S3), which concluded that PRE is an efficient free radical scavenger and provides a protective effect against oxidative damage in a cellular model. Enzymatic antioxidants, such as GPx and SOD, are considered effective in balancing oxidant–antioxidant properties. GPx is responsible for eradicating H_2_O_2_ and lipid hydroperoxides via the reduction–oxidation process. At the same time, SOD participates in the conversion of the main reactive oxygen species (ROS) formed from a variety of sources, most notably, the radical element superoxide (O2•) to hydrogen peroxide (H_2_O_2_) [34].

Following treatment with PRE, the levels of TAS, ORAC, GPx and SOD activities observed in the erythrocytes significantly increased, as demonstrated by the current study’s findings. PRE’s ability to maintain adequate antioxidant defence shows that the treatment was able to restore the supply of antioxidants and prevent their degradation. The favourable effect of antioxidant efficacy in PRE therapy was due to the enhanced antioxidant capacity when compared to SBH treatment, particularly compared to SBH-LD treatment. Thus, the antioxidant properties of the treatment extract might play a significant role in lowering the number of free radicals in diabetic rat samples by acting synergistically or in a multitargeted manner to modulate ROS generation.

#### 3.2.2. Systemic Antioxidant Defence against Diabetes-Induced Oxidative Stress

**Figure 2:**
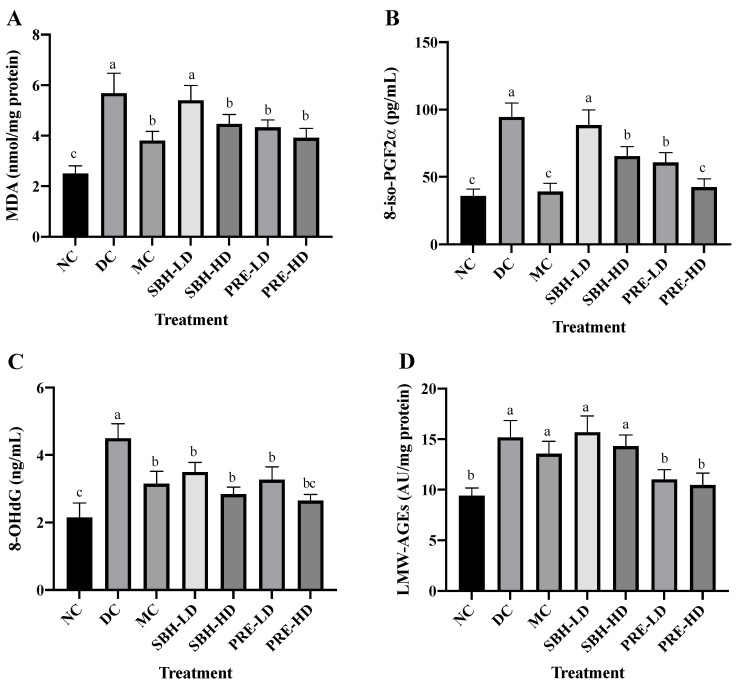
Systemic antioxidant defense against diabetes-induced oxidative stress. The concentrations of serum (**A**) TBARS, (**B**) 8-iso-PGF2α, (**C**) 8-OHdG and (**D**) LMW-AGEs. The values are expressed as the mean ± standard deviation (*n* = 8); different letters indicate significant differences (*p* < 0.05). NC, normal group; DC, diabetic control group without treatment; MC, metformin-treated diabetic control; SBH-LD, diabetic rats treated with stingless bee honey (0.25 g/kg body weight); SBH-HD, diabetic rats treated with stingless bee honey (0.5 g/kg body weight); PRE-LD, diabetic rats treated with the phenolic-rich extract (12.5 mg/kg body weight); PRE-HD, diabetic rats treated with the phenolic-rich extract (25.0 mg/kg body weight).

Oxidative stress-related indicators in rat serum were also analysed to explore the total oxidant–antioxidant balance. Figure 2 depicts the oxidative damage caused to macromolecules, such as lipids, DNA and proteins. The metabolic process triggered by ROS, which causes membrane lipid breakdown, is known as lipid peroxidation. The presence of an excessive concentration of the lipid peroxidation marker TBARS in diabetics indicates an inadequate level of antioxidant protection against ROS-mediated damage. The serum TBARS and 8-iso-PGF2α contents were used to assess lipid oxidative deterioration. The 8-iso-PGF2α compound was produced via a non-enzymatic reaction by ROS on cell membrane arachidonic acid, as the level of 8-iso-PGF2α was frequently used to assess oxidative stress.

The PRE-HD, PRE-LD, SBH-HD and MC groups showed reduced TBARS and 8-iso-PGF2α assays (*p* < 0.05) compared to the DC group, except for SBH-LD, which showed no statistical change compared to the DC group (*p* > 0.05). When compared to the DC group, the PRE-HD group displayed a significant decline in malondialdehyde (MDA) and 8-iso-PGF2α concentration, with corresponding reductions of 31.1% and 55.0% (*p* < 0.05), respectively. There was also no significant difference between the PRE-HD and MC groups and the NC group on 8-iso-PGF2α content. This outcome demonstrated that a concentrated amount of phenolic in the PRE-HD influenced the reduction in lipid peroxidation. This result was consistent with that of Zeni et al. [35], who found that high-phenolic treatment samples also reduced lipid peroxidation in diabetic rats. It was hypothesised that simultaneous increases in the endogenous antioxidant enzyme caused by high phenolic content, which functions as an efficient radical scavenger, would lead to a reduction in lipid peroxidation [36]. Other studies show that the level of MDA was reduced by their respective honey treatments: (1) Gelam honey by Sahhugi et al. [37]; (2) Tualang honey by Erejuwa et al. [33]; and (3) multifloral honey by Busserolles et al., [38] significantly reduced the MDA level in the diabetes-induced rat’s serum.

The oxidative damage to DNA was measured by using 8-OHdG, which acts as a biomarker (Figure 2). There were no significant differences between the PRE-LD, MC, SBH-HD and SBH-LD treatment groups, but all were significantly lower (*p* < 0.05) in the 8-OHdG level when compared to the DC group, which indicated a massive elevation in the DNA’s oxidative stress in the DC group. The serum concentrations of LMW-AGEs were used to evaluate oxidative stress at the protein levels. LMW-AGEs serve as AGE alteration indicators and have a significant hazard potential due to their free interaction with AGE receptors [39]. The PRE-treated groups had lower LMW-AGE concentrations than the DC group, with 31.0% and 27.4% lower concentrations for high and low doses (*p* < 0.05), respectively. Both SBH groups could not lower the LMW-AGE concentration even with the high phenolic equivalent with PRE compared with the DC group (*p* > 0.05), which may be due to the sugar content in the SBH, which interferes with the LMW-AGE’s reduction. However, further analysis needs to be conducted to confirm this statement. There were no significant differences between the PRE treatment and the NC group (*p* > 0.05), with the NC group lowering the LMW-AGE’s concentration by 38.1% compared to the DC group.

In diabetes, hypoinsulinemia increases fatty acyl coenzyme-A oxidase activity, which initiates the β-oxidation of fatty acids, resulting in lipid peroxidation. Additionally, free radicals are produced as a by-product of protein glycation and glucose auto-oxidation, which can then trigger lipid peroxidation [30,40]. High lipid peroxidation diminishes membrane fluidity, which alters the function of membrane-bound enzymes and receptors, impairing membrane function [30,41]. Elevated lipid peroxidation and a deficiency in the antioxidant system have been described as oxidative stress indicators [42]. Based on the data collected, it appears that both doses of PRE and SBH-HD may be capable of providing a protective effect on cell membranes and lipoproteins against the damage caused by lipid peroxides.

Huh et al. [43] reveal a relationship between an increase in 8-OHdG level and the prospect of insulin resistance, which leads to T2DM. From these results, a reduction in serum 8-OHdG following treatment with PRE and SBH implies an enhancement in lowering the DNA’s oxidative stress. Protein oxidative changes are the product of the oxidation in cellular proteins due to stimulation by reactive oxygen [44]. According to Gao et al. [45], glycosylated and oxidised proteins can trigger inflammatory responses due to their interactions with AGE receptors. As a result, reduced levels of AGEs in both PRE-HD and PRE-LD rats imply a lowered risk of oxidative stress in the STZ-NAM-induced rats. Together, these findings show that PREs and a high dose of SBH can recover the overall equilibrium between the oxidative and antioxidative systems in STZ-NAM-induced diabetic rats.

### 3.3. Tissue-Targeted Antioxidant Defence

**Figure 3:**
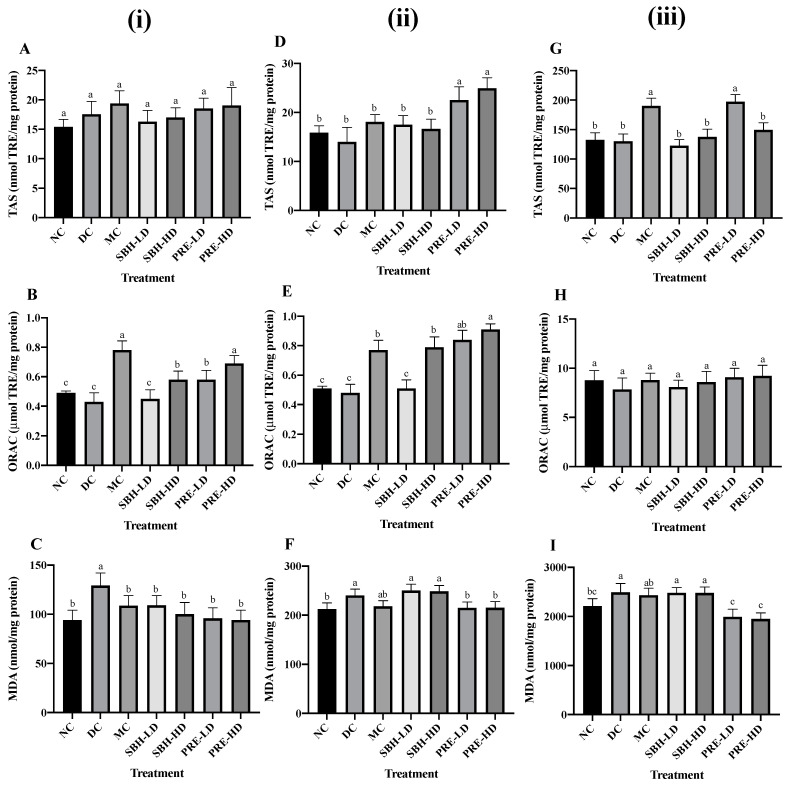
Antioxidant defence on (**i**) liver tissue; (**A**) TAS, (**B**) ORAC, (**C**) TBARS; (**ii**) skeletal muscle tissue; (**D**) TAS, (**E**) ORAC, (**F**) TBARS; (**iii**) adipose tissue; (**G**) TAS, (**H**) ORAC, (**I**) TBARS. The values are expressed as the mean ± standard deviation (*n* = 8); different letters indicate significant differences (*p* < 0.05). NC, normal group; DC, diabetic control group without medication; MC, metformin-treated diabetic control; SBH-LD, diabetic rats treated with stingless bee honey (0.25 g/kg body weight); SBH-HD, diabetic rats treated with stingless bee honey (0.5 g/kg body weight); PRE-LD, diabetic rats treated with the phenolic-rich extract (12.5 mg/kg body weight); PRE-HD, diabetic rats treated with the phenolic-rich extract (25.0 mg/kg body weight).

The liver, skeletal muscle and adipose tissues were the primary target organs for insulin resistance in T2DM [46]. Hence, the antioxidant defence was investigated in these targeted tissues. The KEAP1-NRF2 regulatory pathways were chosen to deeply analyse the transcriptional expression of genes associated with oxidative stress in these tissues. Figure 3 shows the tissue-specific antioxidant and oxidative stress responses, such as TAS, ORAC and TBARS.

For liver tissue antioxidant defence, the data suggest a rise in TAS value for all treatment samples compared to the DC group despite no significant difference among all groups (*p* > 0.05), with the MC group showing the highest TAS value at 19.37 nmol TRE/mg protein. The drug treatment also recorded the highest reading in ORAC, followed by PRE-HD, both of which substantially increased by 81.4% and 60.5%, respectively, (*p* < 0.05) compared to the DC group, indicating that PRE-HD treatment increased the ability of the liver to eradicate AAPH-derived peroxyl radical.

For lipid peroxidation, the DC group showed the highest value of TBARS (*p* < 0.05) compared to all treatment groups. The TBARS value for SBH-LD, SBH-HD, PRE-LD ad PRE-HD were 15.6%, 22.5%, 25.8% and 27.1% lower (*p* < 0.05) compared to the DC group. Another study involving honey treatment on STZ-induced diabetic rat hepatic tissue was reported by Gholami et al. [47], using Jujube honey from Iran. Honey administration reduced the MDA level and increased the TAS level in the diabetic rats after 21 days compared to the untreated diabetic control.

The value of TAS, ORAC and TBARS were evaluated to study the effects of the treatment group on the oxidative stress of skeletal muscle in STZ-NAM-induced diabetic rats. In the finding, PRE-HD improved the antioxidant defence in skeletal muscles to 78.1% (TAS) and 89.6% (ORAC) (*p* < 0.05) compared to the DC group. Surprisingly, both SBH dose treatments indicated the high lipid peroxidation (TBARS) MDA value (SBH-HD; 248.46 nmol/mg) and (SBH-LD; 250.31 nmol/mg) in skeletal tissue along with the untreated diabetic group (240.11 nmol/mg). In contrast, both PRE treatments recorded significantly lower (*p* < 0.05) MDA in skeletal muscle in comparison to the DC group. This result indicates better management of lipid peroxidation in PRE, which involves a concentrated amount of phenolic in comparison to SBH.

The TAS readings of the adipose tissue lysates from all treatment groups did not change significantly (*p* > 0.05), except for the PRE-LD and MC groups. Both groups had elevated TAS values of 51.8% and 46.2%, respectively, compared to the DC group. However, there were no significant differences in the ORAC readings across all groups, including the NC group. Although the MC group showed a marked decrease in MDA concentration by 2.3% compared to the DC group, it was not statistically different (*p* > 0.05). However, both PRE treatments showed a substantial decrease (*p* < 0.05) in lipid peroxidation in adipose tissue in comparison to the DC group. Hemmati et al., [48] reported a major increase in adiponectin concentrations and a significant decrease in MDA level in a local honey treatment on STZ-induced Winstar rats compared to the untreated diabetic rats. Adiponectin hormone, which modulates glucose and lipid regulation, was secreted by adipose tissue and was markedly reduced in diabetic individuals [49].

### 3.4. Gene Expressions on Selected Tissue

**Figure 4:**
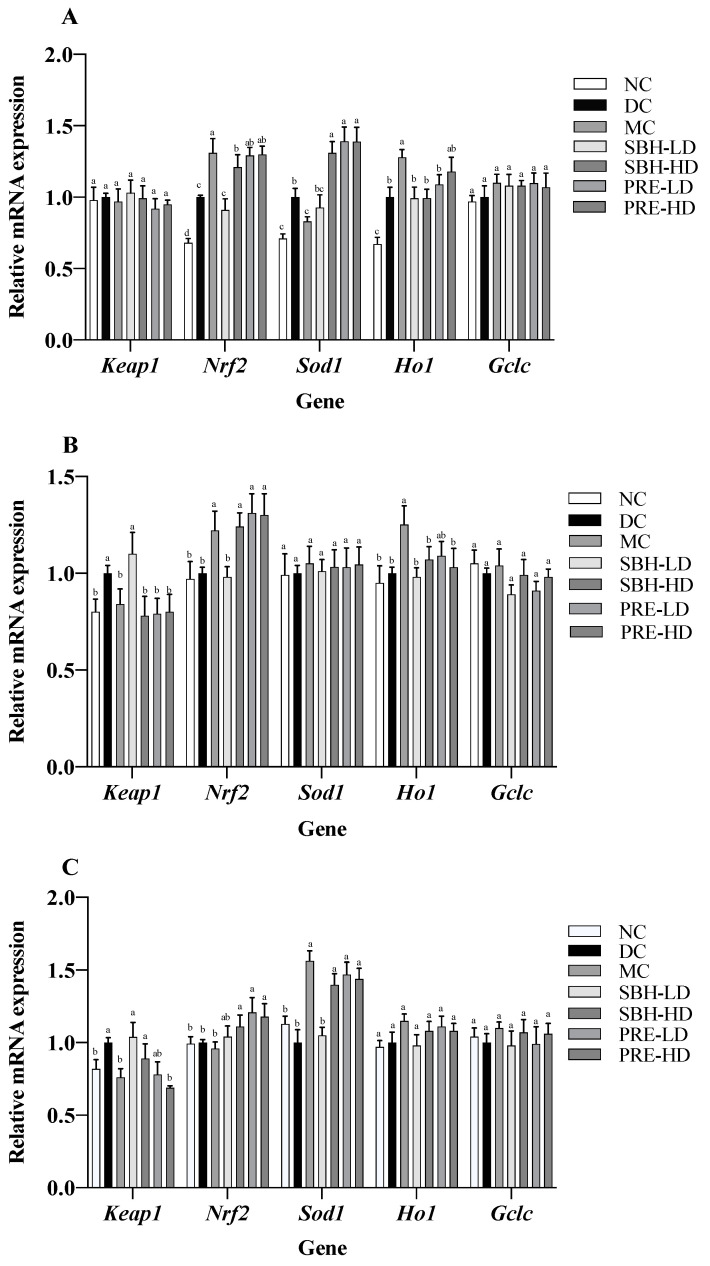
Antioxidant gene expressions in selected tissues: (**A**) liver; (**B**) skeletal muscle; (**C**) adipose tissue. The values are expressed as the mean ± standard deviation (*n* = 8); different letters indicate significant differences (*p* < 0.05) relative to the DC group. NC, normal group; DC, diabetic control group without medication; MC, metformin-treated diabetic control; SBH-LD, diabetic rats treated with stingless bee honey (0.25 g/kg body weight); SBH-HD, diabetic rats treated with stingless bee honey (0.5 g/kg body weight); PRE-LD, diabetic rats treated with the phenolic-rich extract (12.5 mg/kg body weight); PRE-HD, diabetic rats treated with the phenolic-rich extract (25.0 mg/kg body weight).

The expression of several cytoprotective genes, including Ho1, Sod1 and Gclc, was stimulated by the transcription factor Nrf2 [50]. It was established that the activation of the Nrf2 gene can reduce the risk of oxidative liver damage, inflammation, and apoptosis [51]. Subsequent examination of the mRNA expression associated with the KEAP1-NRF2 signalling pathway indicated the upregulation of Nrf2 and Ho1 genes in the PRE-HD and MC groups by 1.3- and 1.3-fold (Nrf2) and by 1.3- and 1.2-fold (Ho1) (*p* < 0.05), respectively, in comparison to the respective DC group (Figure 4). Both high-dose treatment groups also upregulated the expression of Sod1 by 1.4-fold (*p* < 0.05) compared to that in the DC group. However, there were no significant differences (*p* > 0.05) in the expression of Keap1 and Gclc genes across all groups. These results suggest that the enhanced expression of Nrf2, Sod1 and Ho1 may be responsible for the beneficial effects of PRE intervention on antioxidant defence in STZ-NAM-induced diabetic rats. In addition, there was a positive correlation between the TAS with Nrf2 and the TAS with Ho1, with r = 0.96 and r = 0.99, *p* < 0.05, respectively, indicating that the high antioxidant potential in the PRE-HD might influence the upregulation of these genes (Supplementary Figure S2).

Both doses of PRE of stingless bee sample intervention provided a solid indication of a reduction in oxidative stress in skeletal muscle cells, as evidenced by the upregulation (*p* < 0.05) of *Nrf2* and the downregulation (*p* < 0.05) of *Keap1* genes, as well as an increase in the number of TAS and ORAC and a decrease in tissue lipid peroxidation. As Nrf2 is modulated by Keap1 presence, its suppression potential activates Nrf2 and its downstream antioxidant response element [52]. When compared to the DC group, the SBH-HD treatment group showed a 1.2-fold upregulation of Nrf2 and a 0.8-fold downregulation of Keap1 genes (*p* < 0.05). Both PRE treatment groups showed an increase in their *Nrf2* regulation by 1.0-fold compared to the DC group. Also, the metformin-treated group showed elevated expression of *Hod1* by 1.3-fold compared to the DC group. However, there were no significant differences (*p* > 0.05) in *Sod1* and *Gclc* expression across all groups.

The mRNA expression of *Keep 1, Nrf2, Sod1, Ho1* and *Gclc* was analysed in STZ-NAM-induced rats adipose tissue lysate using the KEAP1-NRF2 pathway. Compared to the DC group, the doses of PRE treatment, SBH-HD and MC groups showed significantly higher (*p* < 0.05) *Sod1* expression. *Keap1* was significantly downregulated (*p* < 0.05) in PRE-HD, MC and NC groups by 0.8-, 0.7- and 0.8-fold, respectively, compared to the DC group. On the other hand, there was a significant upregulation (*p* < 0.05) in both PRE treatment groups for the *Nrf2* gene by 1.1- and 1.2-fold, respectively, compared to the DC group. According to these findings, the improved lipid peroxidation in the PRE sample might be attributed to an increase in the downstream antioxidant response *Sod1*, which is triggered by the activation of the NRF2 protein. The substantial correlation between TAS concentration and *Nrf2* gene expression (r = 0.93; *p* < 0.05) demonstrates that a high antioxidant content directly influences antioxidant gene expression (Supplementary Figure S2).

The Keap1-Cul3-E3 ubiquitin ligase complex keeps *Nrf2* at a low level under normal conditions. Cys residues of *Keap1* are oxidised during cellular redox disequilibrium, resulting in a conformational shift in the complex that releases NRF2, which subsequently translocates to the nucleus [53]. An increase in *Nrf2* expression promotes an increase in the expression of different downstream antioxidant responses, such as *Sod1, Ho1* and *Gclc*, which can be observed primarily in both doses of PRE treatment as well as SBH-HD treatment in comparison with non-treated diabetic rats. The upregulation of *Nrf2* genes with PRE-HD, PRE-LD and SBH-HD treatment was also consistent in all targetted tissues in comparison to the DC group. In addition, several *Nrf2* activator compounds have been shown to engage directly with the adipose tissue (in vivo) in the prospect of being therapeutic options for diabetes, such as β-cryptoxanthin and sulforaphane (Sprague Dawley model), hydroxy-tyrosol and glucoraphanin (C57BL/6J mice), and *Salvia hispanica L*. (Winstar rat) [54]. The current study’s findings showed that PRE and SBH-HD treatment might be able to modulate the antioxidant response in experimentally diabetic rats by targeting the KEAP1-NRF2 pathway.

### 3.5. Protein Translation on Hepatic Tissue

**Figure 5:**
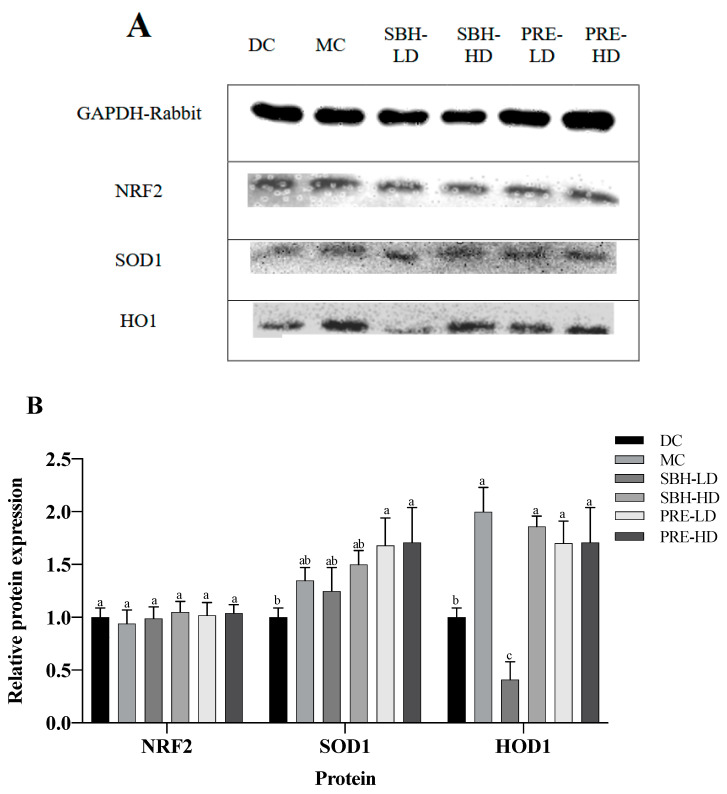
Protein expression in the liver tissue. (**A**) Total liver tissue lysate with GAPDH housekeeping; (**B**) relative targeted protein expression. The values are expressed as the mean ± standard deviation (*n* = 8); different letters indicate significant differences (*p* < 0.05) relative to the DC group. NC, normal group; DC, diabetic control group without medication; MC, metformin-treated diabetic control; SBH-LD, diabetic rats treated with stingless bee honey (0.25 g/kg body weight); SBH-HD, diabetic rats treated with stingless bee honey (0.5 g/kg body weight); PRE-LD, diabetic rats treated with the phenolic-rich extract (12.5 mg/kg body weight); PRE-HD, diabetic rats treated with the phenolic-rich extract (25.0 mg/kg body weight).

The hepatic protein expression of NRF2, SOD1 and HO1 is shown in Figure 5, with GAPDH used as a housekeeping protein. Both PRE treatments elevated the expression of SOD1 in comparison to the DC group (*p* < 0.05). There was also upregulated translation of HO1 protein in the PRE-HD, PRE-LD and SBH-HD groups compared to the DC group by 1.71-, 1.70- and 1.86-fold (*p* < 0.05). However, there were no significant differences (*p* > 0.05) in the expression of the protein NRF2 across all treatment groups compared to the DC group.

The NRF2, HO1 and SOD1 expressions were measured to understand the protective effect of PRE-treated STZ-NAM-induced diabetic rats against ROS. NRF2 controls the regulation of cytoprotective genes to preserve cellular homeostasis under stressful conditions. Under high oxidative stress conditions, NRF2 dissociates from KEAP1 and stimulates the expression of antioxidant genes and proteins by binding to an antioxidant response element (ARE) in the nucleus [53,55]. A high dose of SBH successfully elevated the downstream antioxidative response of the HO1 protein, but not at a low dose of SBH treatment. In contrast, both doses of PRE treatment elevated the antioxidative proteins HO1 and SOD1 (*p* < 0.05), suggesting that the treatment activated the NRF2 pathway, which protects the liver from oxidative damage produced by STZ-NAM-induced diabetes.

## 4. Conclusions

In conclusion, the findings indicate that SBH and PRE treatment in high-fat diet-fed STZ-NAM-induced diabetic rats had beneficial effects on the oxidant–antioxidant balance without compromising liver and kidney functions. Furthermore, both doses of PRE and SBH-HD improved systemic oxidant–antioxidant equilibrium, with varied positive effects on the liver, adipose and muscle tissues. The observed transcriptional alterations in antioxidant genes suggest a tissue-specific modulation of the KEAP1-NRF2 signalling pathways in response to SBH and PRE treatment. These results strongly suggest that the phenolic compounds present in SBH and PRE play a crucial role in enhancing overall and systemic antioxidant defenses, as evidenced by the improved expression of antioxidant genes and proteins in the high-fat-fed STZ-NAM-induced diabetic rat model. Collectively, this study highlights the potential of SBH and PRE as therapeutic agents for restoring the oxidant–antioxidant imbalance associated with diabetes, offering insights into their tissue-specific effects and the underlying molecular mechanisms. Further research is warranted to explore the full therapeutic potential of these natural compounds in the context of diabetes and oxidative stress-related disorders.

## Figures and Tables

**Table 1:** Animal treatment groups.

Animal	Description	Treatment
Group		
NC	Normal control (non-diabetic)	Distilled water
	Normal pellet diet-fed	
DC	Diabetes control without medications	Distilled water
	High fat diet-fed	
	STZ-NAM-induced diabetes	
MC	Metformin-treated diabetes control	Metformin
	High-fat diet-fed	(10.0 mg/kg body weight)
	STZ-NAM-induced diabetes	
SBH-LD	Diabetic test rats	Stingless bee honey (low dose)
	High-fat diet-fed	(0.25 g/kg body weight)
	STZ-NAM-induced diabetes	
SBH-HD	Diabetic test rats	Stingless bee honey (high dose)
	High-fat diet-fed	(0.5 g/kg body weight)
	STZ-NAM-induced diabetes	
PRE-LD	Diabetic test rats	Phenolic-rich extract (low dose)
	High-fat diet-fed	(12.5 mg/kg body weight)
	STZ-NAM-induced diabetes	
PRE-HD	Diabetic test rats	Phenolic-rich extract (high dose)
	High-fat diet-fed	(25.0 mg/kg body weight)
	STZ-NAM-induced diabetes	

**Table 2:** Biochemical parameter.

Treatment								
	Unit	NC	DC	MC	SBH-LD	SBH-HD	PRE-LD	PRE-HD
**Biochemical** **Parameter (Serum)**								
AST	U/L	268.01 ± 26.00 ^c^	328.82 ± 38.11 ^a^	296.67 ± 26.12 ^b^	311.11 ± 31.02 ^ab^	307.54 ± 33.79 ^b^	301.24 ± 29.08 ^b^	299.37 ± 19.19 ^b^
ALP	U/L	164.50 ± 22.11 ^c^	279.44 ± 22.62 ^a^	200.01 ± 12.23 ^b^	241.20 ± 12.29 ^ab^	216.34 ± 14.71 ^b^	216.06 ± 20.11 ^b^	172.29 ± 11.92 ^c^
ALT	U/L	94.94 ± 16.72 ^c^	156.81 ± 29.11 ^a^	96.62 ± 15.74 ^c^	130.10 ± 15.77 ^b^	120.51 ± 11.14 ^b^	109.33 ± 13.26 ^c^	93.43 ± 10.07 ^c^
GGT	U/L	11.83 ± 2.53 ^a^	12.11 ± 2.32 ^a^	12.45 ± 1.42 ^a^	12.19 ± 1.73 ^a^	11.99 ± 3.26 ^a^	12.87 ± 2.15 ^a^	12.64 ± 1.74 ^a^
Urea	mmol/L	4.42 ± 1.49 ^a^	5.61 ± 0.64 ^a^	5.07 ± 0.60 ^a^	5.29 ± 1.12 ^a^	5.41 ± 1.17 ^a^	5.48 ± 1.33 ^a^	5.01 ± 1.18 ^a^
Creatinine	μmol/L	39.50 ± 3.38 ^b^	53.81 ± 1.42 ^a^	47.86 ± 2.12 ^a^	49.84 ± 2.82 ^a^	47.85 ± 3.90 ^a^	47.19 ± 8.12 ^a^	42.72 ± 7.43 ^b^

The values are expressed as the mean ± standard deviation (*n* = 8); different letters indicate significant differences (*p* < 0.05) across the rows. NC, normal group; DC, diabetic control group without treatment; MC, metformin-treated diabetic control; SBH-LD, diabetic rats treated with stingless bee honey (0.25 g/kg body weight); SBH-HD, diabetic rats treated with stingless bee honey (0.5 g/kg body weight); PRE-LD, diabetic rats treated with the phenolic-rich extract (12.5 mg/kg body weight); PRE-HD, diabetic rats treated with the phenolic-rich extract (25.0 mg/kg body weight).

## Data Availability

Not applicable.

## Supplementary Materials

The following supporting information can be downloaded at: https://www.mdpi.com/article/10.3390/nu15132835/s1, Figure S1: Weekly body weight changes of treated rats; Figure S2: Correlation heat map of (A) liver, (B) skeletal muscle and (C) adipose tissue mRNA expression and antioxidant defence parameters; Figure S3: HPLC profiles of phenolic compounds of PRE detected at 280 nm; Table S1: The nucleotide sequences of primers used in qPCR.
