# Supplementary material for: Stingless Bee (Heterotrigona Itama) Honey and Its Phenolic-Rich Extract Ameliorate Oxidant–Antioxidant Balance via KEAP1-NRF2 Signalling Pathway
Source: Nutrients. 2023 Jun 22;15(13):2835. doi: 10.3390/nu15132835 (PMC10343317; doi:10.3390/nu15132835)
Supplement: Supplementary file 1 [file nutrients-15-02835-s001.zip › nutrients-2450948-supplementary.pdf]

Supplementary materials

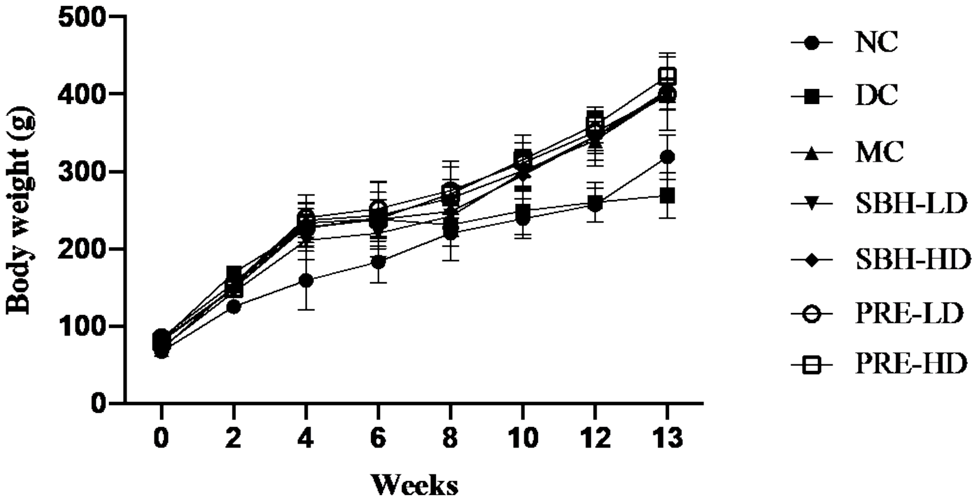

Supplementary Figure S1. Weekly body weight changes of treated rats. The values are expressed as the mean ± standard deviation (n = 8); HFD, high-fat diet fed start; STZ-NAM, streptozotocin-nicotinamide induction; NC, normal group; DC, diabetic control group without medication; MC, metformin-treated diabetic control; SBH-LD, diabetic rats treated with stingless bee honey (0.25 g/kg body weight); SBH-HD, diabetic rats treated with stingless bee honey (0.5 g/kg body weight); PRE-LD, diabetic rats treated with the phenolic-rich extract (12.5 mg/kg body weight); PRE-HD, diabetic rats treated with the phenolic-rich extract (25.0 mg/kg body weight)

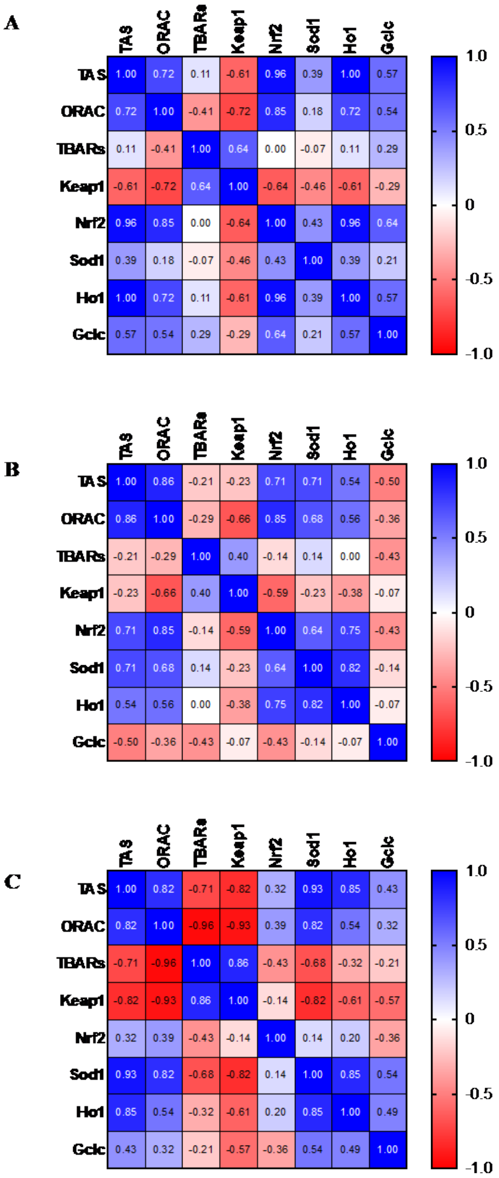

Supplementary Figure S2. Correlation heat map of (A) liver, (B) skeletal muscle and (C) adipose tissue mRNA expression and antioxidant defence parameters. Each cell indicates the Spearman correlation coefficient value, r, for a pair of mRNA expression and antioxidant defence parameters. Positive correlations ($0 < r < 1.0$) are displayed in blue colour and negative correlations ($-1.0 < r < 0$) are displayed in red colour. Colour intensity is proportional to the correlation coefficients.

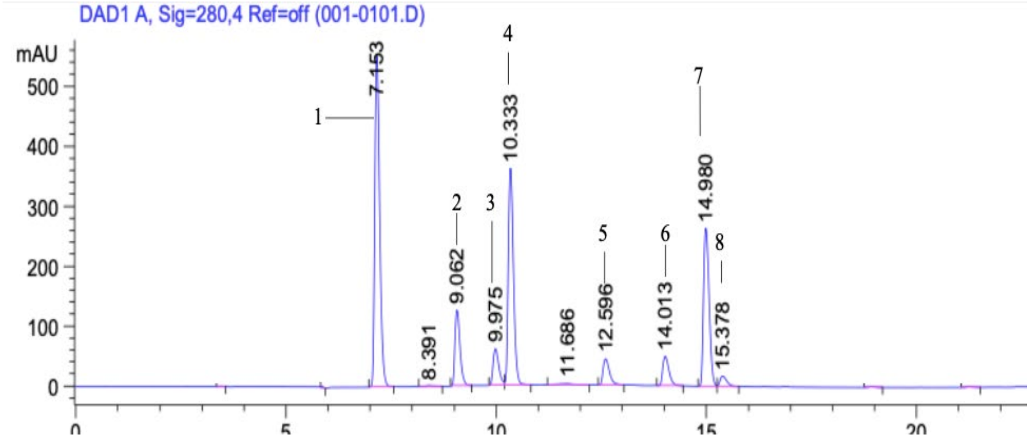

Supplementary Figure S3. HPLC profiles of phenolic compounds of PRE detected at 280 nm. Chromatograms: gallic acid (1), epicatechin (2), chlorogenic acid (3), myricetin (4), syringic acid (5), quercetin (6), kaempferol (7), cinnamic acid (8).

Dosage calculation

% of yield	= 2 g SBH yield 100 mg (average of 99.77 mg average) PRE
	= 100 mg PRE/ 2000 mg SBH x 100% = 5% yield
SBH-HD =	= 0.5 g/kg
If 2g SBH yielded 100 mg PRE	= 0.5 g/ 2 g x 100 % = 25 mg/kg PRE-HD
Therefore	= 0.5 g/kg SBH-HD equivalent to 25 mg/kg PRE-HD
SBH-LD =	= 0.25 g/kg
If 2g SBH yielded 100 mg PRE	= 0.25 g/ 2g x 100% = 12.5 mg/kg PRE-HD
Therefore	= 0.25 g/kg SBH-LD equivalent to 12.5 mg/kg PRE-LD

List of primer sequences

Supplementary Table S1. The nucleotide sequences of primers used in qPCR

Genes	Accession number	Forward primers (3′-5′)	Reverse primers (5′-3′)
Keap1	NM_057152	AGGTGACACTATAGAATA CTACAACCCGAGCAATG	GTACGACTCACTATAGGGAC TGGCTCATATCTCTCCA
Nrf-2	NM_006164.4	AGGTGACACTATAGAATATCG CAACAACTCTTTATCT	GTACGACTCACTATAGGGAA GAGGAGGTCTCCGTTA
Ho1	NM_002133.2	AGGTGACACTATAGAATAACT GCGTTCCTGCTCAACAT	GTACGACTCACTATAGGGAG GGCAGAATCTTGCACTTTGT
Sod1	NM_000454.4	AGGTGACACTATAGAATAAA GTACAAAGACAGGAAACG	GTACGACTCACTATAGGGATG ACAAGTTTAATACCCATCT
Gclc	NM_012815	AGGTGACACTATAGAATAA ATGTGCCAATATTCAAGG	GTACGACTCACTATAGGGAAT AAAGGTATCTTGCCTCAG
*Gapdh**	NM_001357943.2	GTCATCCCTGAGCTGAACGG	CCACCTGGTGCTCAGTGTAG

Based on Homo sapiens gene sequences adopted from National Centre for Biotechnology Information GenBank Database (NCBI)

*Housekeeping gene
